# Airflow-Restricting Mask Reduces Acute Performance in Resistance Exercise

**DOI:** 10.3390/sports4040046

**Published:** 2016-09-23

**Authors:** Yuri L. Motoyama, Gustavo B. Joel, Paulo E. A. Pereira, Gilmar J. Esteves, Paulo H. S. M. Azevedo

**Affiliations:** 1 Group of Studies and Research in Exercise Physiology (GEPEFEX), Federal University of São Paulo, São Paulo 11.015-020, Brazil; pereira.pauloeduardo@hotmail.com (P.E.A.P.); gilmaresteves@hotmail.com (G.J.E.); 2 Institute of Physical Activity Sciences and Sport, Cruzeiro do Sul University, São Paulo 03.342-000, Brazil; gustavo_barquilha@hotmail.com; 3 Postgraduate Program in Human Movement Sciences and Rehabilitation, Group of Studies and Research in Exercise Physiology (GEPEFEX), Federal University of São Paulo, São Paulo 11.015-020, Brazil; paulohsmazevedo@gmail.com

**Keywords:** lactate, strength training, central fatigue, lactate paradox

## Abstract

Background: The aim of this study was to compare the number of repetitions to volitional failure, the blood lactate concentration, and the perceived exertion to resistance training with and without an airflow-restricting mask. Methods: Eight participants participated in a randomized, counterbalanced, crossover study. Participants were assigned to an airflow-restricting mask group (MASK) or a control group (CONT) and completed five sets of chest presses and parallel squats until failure at 75% one-repetition-maximum test (1RM) with 60 s of rest between sets. Ratings of perceived exertion (RPEs), blood lactate concentrations (Lac^−^), and total repetitions were taken after the training session. Results: MASK total repetitions were lower than those of the CONT, and (Lac^−^) and MASK RPEs were higher than those of the CONT in both exercises. Conclusions: We conclude that an airflow-restricting mask in combination with resistance training increase perceptions of exertion and decrease muscular performance and lactate concentrations when compared to resistance training without this accessory. This evidence shows that the airflow-restricting mask may change the central nervous system and stop the exercise beforehand to prevent some biological damage.

## 1. Introduction

In a common sense, resistance training should be as hard as possible to induce a high level of hypertrophy and muscular endurance. Training to failure, forced repetition, vascular occlusion are recognized methods used to improve the physiological responses during training sessions [1]. With the Olympic Games in 1968, many coaches and researches turned their attention to high-altitude exposure effects on performance [2]. Since then, researches have tried to use strategies such as training at sea level and resting at altitude to obtain benefits associated with this methodology [3].

An accessory called the Elevation Training Mask 2.0 (Training Mask, Cadillac, MI, USA) was developed to simulate the effects of altitude during physical exercises through valves that restrict airflow. Recently, athletes (Mixed Martial Arts fighters, cyclists, and runners) have been using an airflow-restricting mask during their training—including resistance exercise—believing that this new method induces hypoxemia and a high metabolic response. Sellers and his group [4] found no differences in chronic responses between aerobic and anaerobic training groups using an airflow-restricting mask. Porcari [5] found improvements associated only with inspiratory muscles training with altitude simulation effects in aerobic exercises. However, the literature needs data to clarify the prescription of an airflow-restricting mask in resistance exercises.

The aim of this study was to compare the number of repetitions (acute performance), the rating of perceived exertion (central nervous system), and the blood lactate concentration (metabolic) between sessions of resistance training with and without an airflow-restricting mask. Our hypothesis is that the accessory can reduce performance (repetitions) and increase perceived exertion and blood lactate concentration.

## 2. Materials and Methods

### 2.1. Participants

Eight resistance-trained men (age 26.9 ± 2.2 years; height 177.9 ± 3.0 cm; body mass 85.4 ± 3.0 kg; % body fat 14.0 ± 1.2, mean ± SD) participated in this study. The participants included had a minimum resistance training experience of six months and 3 sessions per week were included and had no pharmacological ergogenic strategy. Participants had no muscle injury that would compromise the study. Participants who did not respect the interval between tests (rest and frequency) were excluded. All participants were informed of the procedures and potential risks before testing and signed the consent form. The study is in accordance with the Code of Ethics of the World Medical Association (Declaration of Helsinki) and the local ethics committee (number 1.172.298). All participants were instructed to repeat a pattern of meal and water intake prior to testing.

### 2.2. Protocol

Participants performed 5 sets to volitional failure at 75% one-repetition-maximum test (1RM) with bench press and squat exercises consecutively in two different groups: one group with an airflow-restricting mask (MASK) and one without one (CONT) (a randomized, counterbalanced, crossover study). The interval between series of repetitions was 60 s, and the order of exercises was randomized. The speed was controlled by an electronic metronome, allowing 2 s for concentric and eccentric phases. The bench press was performed with maximum amplitude and squats to reach 90 degrees of knee flexion. Participants had three days of rest between tests (in both CONT and MASK).

The 1RM test assessment was made after one minute of warm-up, which consisted of 2 sets of 10 repetitions with a light load (subjective perception) and 60 s of rest. The selection of initial load was based on the participant's experience. An interval of 3 min between attempts and exercises was provided. The test ended when the participant could not perform the complete movement.

### 2.3. Airflow Restricting Mask Procedures

The wearing of the airflow-restricting mask was after 15 min of total rest and completely covering the mouth and nostrils. The control of air intake through mask valves was identical for all participants. The activated resistance level was set to 18× (measure of resistance-air value proposed by accessory developers).

### 2.4. Rating of Perceived Exertion

The measurement of RPE was made immediately before the rest and after five sets of each exercise. The scale used was 1 to 10 arbitrary units on the Borg CR-10 scale [6].

### 2.5. Lactate Concentration

Immediately before the experiment and after the end of 5 sets of bench press and parallel squats, blood was drawn to measure lactate concentration. Blood lactate levels were measured using a portable lactometer (Accutrend Plus^®^, Accusport, Hawthorne, NY, USA) from the earlobe.

### 2.6. Heart Rate

Heart rate was measured with a portable monitor (Polar^®^rs800x, Oulu, Finland) during all of 5 sets of bench presses and parallel squats (MASK and CONT), and this data is shown as the mean of all sets.

### 2.7. Statistical Analysis

To examine the normal distribution of data, we used the Shapiro–Wilk test, and data were considered normal. A paired T-test was applied to detect differences between CONT and MASK. Possible degrees of violation in sphericity was corrected by a Mauchly W test and an analysis of variance with repeated measures (ANOVA). The Bonferroni post-hoc was then applied for comparison between sets. An alpha level of *P* ≤ 0.05 and beta level of 80% was considered acceptable. A coefficient of variation (CV) was shown to standardize the measure of dispersion [7]. To calculate the effect size (ES), the Hedge’s g approach was used, and data is shown with their respective 95% confidence intervals (CIs) [8]. To classify the ES, we used a qualitative scale developed by Cohen adapted by Rosenthal [9]; to estimate the probability of the superior outcome of one treatment over another, we used the common language effect size statistic [10].

## 3. Results

Acute performance (number of repetitions) for upper and lower limbs was different between both conditions for all 5 sets. All ES data were classified as very large comparing MASK and CONT average repetitions in parallel squats (ES = 2.16 (CI = −2.74 to −1.50)) and chest presses (ES = 1.85 (CI = −2.41 to 1.29)).

The average value of repetitions for the parallel squats was lower for the MASK than for the CONT (10.48 ± 1.33; CV = 12% and 13.30 ± 1.19; CV = 8%; *P* = 0.0001). This result showed that using an airflow-restricting mask could harm muscular performance during a session of resistance exercise when compared with the CONT. Table 1 shows statistical results between sets with *P*-values lower than 0.05. The ES and its respective confidence interval were calculated to strengthen the conclusions based on the *P*-value (within groups) in the CONT for the parallel squats.

The average value of repetitions for the chest presses was lower in the MASK than in the CONT (10.85 ± 0.76; CV = 7% and 13.10 ± 1.43; CV = 10%; *P* = 0.001). Table 2 shows the statistical results between sets with *P*-values lower than 0.05. The ES and its respective confidence interval were calculated to strengthen the conclusions based on the *P*-value (within groups) in the CONT for the chest presses.

A significant increase on RPE, compared to CONT, was observed after airflow-restricting MASK exercise conditions (8.31 ± 0.46; CV = 5% and 5.25 ± 0.71; CV = 13%; *P* = 0.0001; ES = 4.86 (CI = 4.56 to 5.15)).

A significant decrease in post-set blood lactate concentration, compared to the CONT, was observed after MASK exercise conditions (7.38 ± 0.63; CV = 8% and 10.5 ± 2.09; CV = 19%; *P* = 0.006; ES = −1.91 (CI = −2.66 to −1.14)).

Mean heart rate (mHR) in the MASK was higher than that of the CONT (154.5 ± 9.4; CV = 6% and 124.5 ± 2.6; CV = 2%; *P* = 0.0001; ES = 4.08 (CI = 0.67 to 7.48)).

## 4. Discussion

**Figure 1:**
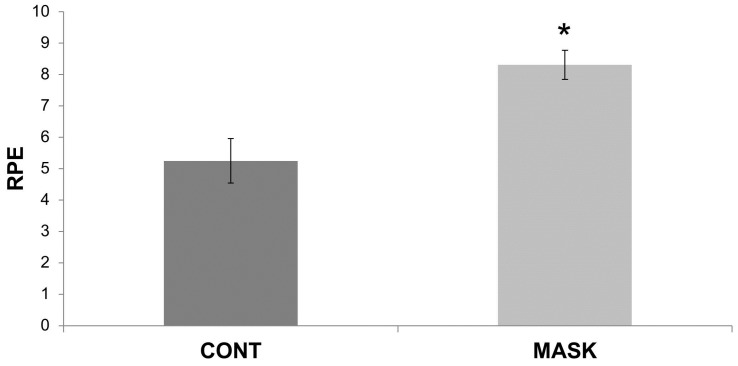
RPE after the control (CONT) and airflow-restricting (MASK) conditions. * different of CONT group.

**Figure 2:**
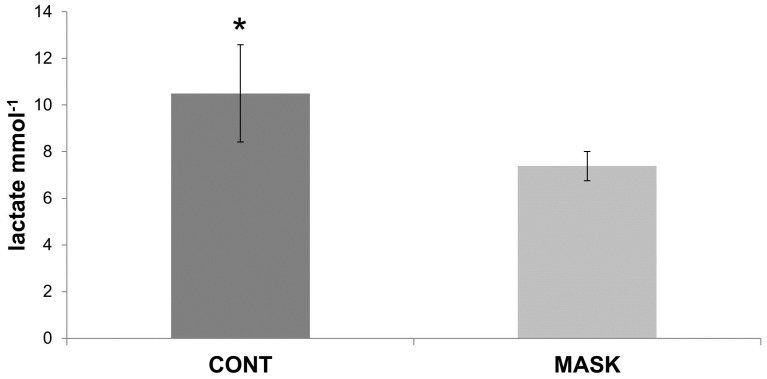
Lactate concentrations after the control (CONT) and airflow-restricting (MASK) conditions. * different of MASK group.

This is the first study that has compared the effects of resistance training with and without an airflow-restricting mask on muscle performance, RPE, and (Lac^−^). The major findings of this study were that resistance training combined with an airflow-restricting training mask acutely increased RPE (Figure 1) and decreased muscular performance and (Lac^−^) (Figure 2), similar to the concept of the lactate paradox [11]. The decrease in muscular performance was independent of limbs.

**Figure 3:**
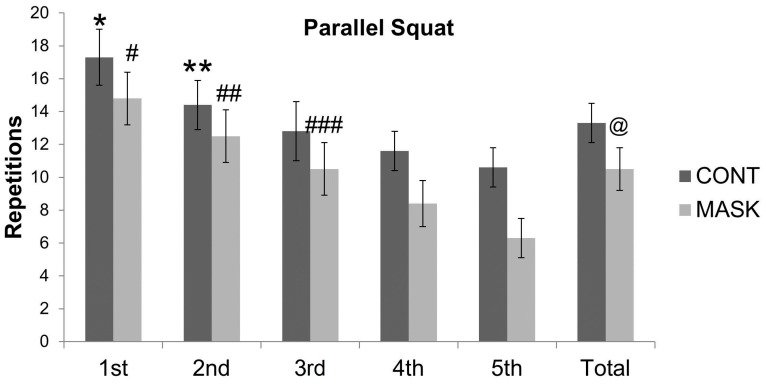
Values are means ± SD. * different from all sets within group; ** different from 1st, 4th, and 5th sets within group; # different from all sets within group; ## different from 1st, 4th, and 5th sets within group; ### different from 1st and 5th sets within group; @ different from the control group. Number of repetitions of parallel squats in the control group (CONT) and the airflow-restricting mask group (MASK). Bars labeled “Total” are the average values of five sets.

**Figure 4:**
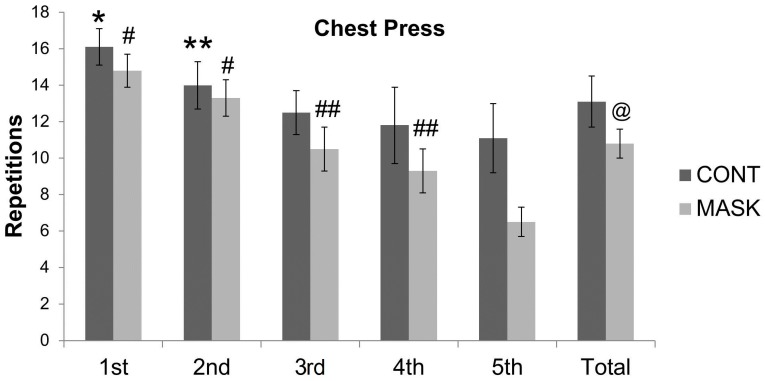
Values are mean s± SD. * different from 3th, 4th and 5th sets within group; ** different from 5th set within group; # different from 3th, 4th and 5th sets within group; ## different from 1st, 2nd and 5th sets within group; @ different from control group. The number of chest press repetitions in the control group (CONT) and the airflow-restricting mask group (MASK). Bars called “Total” are the average values of five sets.

The number of repetitions (i.e., muscular performance) performed during resistance exercise is an important training variable and may modulate blood lactate concentration [12]. The CONT and the MASK showed a decrease in the number of repetitions along the sets (Figure 3 and Figure 4). Additionally, comparing groups, the MASK demonstrated lower total repetitions than the CONT. For parallel squats, the average difference showed a very large ES and a 93% probability that a subject using an airflow-restricting mask would have lower performance than the control group. Observing the mean difference in chest press repetition, we noticed a very large ES and a probability of superiority of 89%. The decrease in the total number of repetitions could have impacted the total time under tension in the session training, which is important for strength gain and hypertrophy in the long term [13]. Therefore, a decrement in repetitions and consequently time under tension might harm the strength and hypertrophy improvement.

We speculate that the highest impairment of MASK may be explained by central fatigue theory (central governor model) because, independent of the real hypoxic condition, the RPE increased after exercise and blood lactate decreased. Lactate is a marker of glycolytic metabolism and intensity of exercise. There is a correlation between blood lactate concentration, exercise intensity, muscular tissue hypoxia, metabolic acidosis, and fatigue [14]. Analysis revealed that the blood lactate concentration in the MASK conditions was lower than that of the control group (Figure 2). A very large ES supports this difference with a 91% probability that a subject using an airflow-restricting mask had lower (Lac^−^) than the control group. According to Lin et al. [15] testosterone release is affected by (Lac^−^) in a dose-dependent manner. This is important for resistance-training goals because testosterone is an anabolic hormone that promotes muscle mass improvement. Other studies have shown that tissue hypoxia during resistance training, such as blood flux restriction (kaatsu training), induces a pH fall and a rise in (Lac^−^] [16]. Therefore, our data are opposite to the traditional heavy-resistance training and kaatsu training method, which promotes acutely high (Lac^−^). This difference might be explained by a phenomenon called the lactate paradox. The paradox lactate is described by the lower blood lactate concentrations during maximal exercise at altitude [11]. The literature does not show any data of this phenomenon through resistance training. In airflow-restricting conditions, it was expected that (Lac^−^) suddenly increases, but this response did not occur. Therefore, resistance training with an airflow-restricting mask does not appear to promote this beneficial physiological response, and the probability of this phenomenon is about 91%.

The RPE is used to indicate how people feel during and after physical exercise, that is, to measure perceptual intensities [17]. It might be influenced by psychological (central factors), cardiorespiratory (e.g., ventilation), and metabolic factors (e.g., blood lactate) in the feed-forward mechanism [18,19,20]. In this study, the RPE observed in the MASK was greater than that of the control group (Figure 1), indicating that RPE may influence the fatigue process. This data becomes stronger when we accept the very large ES beyond the *P*-value (the largest ES of this study) with a probability of superiority close to 100%. The influence of wearing the airflow-restricting mask can be explained considering the unpleasant sensation caused by the accessory. This relation was observed through a relation between brain structures that process the fatigue sensation and performance (e.g., insular cortex) [18,21].

Observing the heart rate, we can see higher post-test values in the MASK than the CONT. This data shows a very large ES and a probability of superiority close to 100%. Noakes [22] established a relationship between a HR rise and a protective (possible) central mechanism that prevents heart ischemia. According to this theory, a HR rise can lead to a consecutive increase of coronary flow independently of cardiac output (physiological body needs). The central command interrupts the exertion to prevent cardiac damage.

Some limitations to this study were the absence of hormonal response analyses (e.g., growth hormone, testosterone, and cortisol), micro-damage biomarkers (e.g., creatine kinase and lactate dehydrogenase), blood oxygen saturation, and electromyography. These physiological and biochemical parameters can improve our understanding of the difference between groups.

## 5. Conclusions

An airflow-restricting mask in combination with resistance exercise increases the perception of exertion and decreases muscular performance and blood lactate concentration when compared to resistance training alone. Therefore, resistance exercise with an airflow-restricting mask provides neither the metabolic response expected nor an advantageous method for resistance training. One hypothesis that explains this decrease in performance is that the low air supply promoted by the airflow-restricting mask influences the central nervous system and stops the exercise from preventing biological damage.

## Figures and Tables

**Table 1:** Parallel squat data description and statistical results. Data are presented by means ± standard deviation (SD); CV = coefficient of variation (express in percentage); ES = effect size; ES-IC = confidence interval of effect size.

Control Group	Mean ± SD			CV (%)			*P* Value	ES	ES-IC		
1º × 2º sets	17.3 ± 1.7	×	14.4 ± 1.5	9	and	12	0.004	–1.71	–2.48	to	–0.93
1º × 3º sets	17.3 ± 1.7	×	12.8 ± 1.7	9	and	13	0.0001	–2.48	–3.32	to	–1.64
1º × 4º sets	17.3 ± 1.7	×	11.6 ± 1.2	9	and	10	0.0001	–3.71	–4.42	to	–3.00
1º × 5º sets	17.3 ± 1.7	×	10.6 ± 1.2	9	and	11	0.0001	–4.36	–5.07	to	–3.65
2º × 4º sets	14.4 ± 1.5	×	11.6 ± 1.2	12	and	10	0.004	–1.94	–2.61	to	–1.28
2º × 5º sets	14.4 ± 1.5	×	10.6 ± 1.2	12	and	11	0.0001	–2.61	–2.54	to	–1.97
**Mask Group**											
1º × 2º sets	14.7 ± 1.6	×	12.5 ± 1.6	10	and	12	0.0001	–1.36	–2.11	to	–0.55
1º × 3º sets	14.7 ± 1.6	×	10.5 ± 1.6	10	and	15	0.0001	–2.58	–3.34	to	1.82
1º × 4º sets	14.7 ± 1.6	×	8.38 ± 1.4	10	and	16	0.0001	–4.12	–4.83	to	–3.40
1º × 5º sets	14.7 ± 1.6	×	6.1 ± 1.1	10	and	18	0.001	–6.03	–6.69	to	–5.37
2º × 4º sets	12.5 ± 1.6	×	8.38 ± 1.4	12	and	16	0.0001	–2.58	–3.32	to	–1.84
2º × 5º sets	12.5 ± 1.6	×	6.1 ± 1.1	12	and	18	0.0001	–4.21	–4.90	to	–3.52
3º × 5º sets	10.5 ± 1.6	×	6.1 ± 1.1	15	and	18	0.0001	–2.86	–3.55	to	–2.18

**Table 2:** Chest Press data description and statistical results. Data are presented by means ± standard deviation (SD); CV = coefficient of variation (express in percentage); ES = effect size; ES-IC = confidence interval of effect size.

Control Group	Mean ± SD			CV (%)			*P* value	ES	ES-IC		
1º × 3º sets	16.1 ± 1.0	×	12.5 ± 1.2	6	and	9	0.0001	–3.12	–3.65	to	–2.58
1º × 4º sets	16.1 ± 1.0	×	11.7 ± 2.1	6	and	18	0.0001	–2.50	–3.31	to	–1.69
1º × 5º sets	16.1 ± 1.0	×	11.1 ± 1.9	6	and	16	0.0001	–3.13	–3.87	to	2.39
2º × 5º sets	14.0 ± 1.3	×	11.1 ± 1.9	9	and	16	0.008	–1.67	–2.47	to	–0.88
**Mask Group**											
1º × 3º sets	14.7 ± 0.9	×	10.5 ± 1.2	6	and	11	0.0001	–3.81	–4.33	to	–3.30
1º × 4º sets	14.7 ± 0.9	×	9.25 ± 1.2	6	and	12	0.0001	–5.03	–5.53	to	–4.52
1º × 5º sets	14.7 ± 0.9	×	6.5 ± 0.8	6	and	11	0.0001	–9.42	–9.83	to	–9.02
2º × 3º sets	13.2 ± 1.0	×	10.5 ± 1.2	7	and	11	0.0001	–3.77	–4.26	to	–3.28
2º × 4º sets	13.2 ± 1.0	×	9.25 ± 1.2	7	and	12	0.0001	–2.64	–3.12	to	–2.16
2º × 5º sets	13.2 ± 1.0	×	6.5 ± 0.8	7	and	11	0.001	–2.32	–2.87	to	–1.77
3º × 5º sets	10.5 ± 1.2	×	6.5 ± 0.8	11	and	11	0.0001	–3.43	–3.97	to	–2.89
4º × 5º sets	9.25 ± 1.2	×	6.5 ± 0.8	12	and	11	0.0001	–7.00	–7.45	to	–6.56
